# How do Alcohol Use and Depression Predict Grip Strength among Middle-Aged and Older Adults?

**DOI:** 10.1093/geroni/igab046.3119

**Published:** 2021-12-17

**Authors:** Song Ge, Linda Dune, Desiree' Frantz, Laurel Laviolette, Mary Njuguna, xianping tang, Changwei Li

**Affiliations:** 1 University of Houston-Downtown, University of Houston-Downtown, Texas, United States; 2 University of Houston Downtown, Houston, Texas, United States; 3 University of Houston downtown, Richmond, Texas, United States; 4 Xuzhou Medical University, Xuzhou, Jiangsu, China (People's Republic); 5 Tulane University School of Public Health and Tropical Medicine, New Orleans, Louisiana, United States

## Abstract

Background: Physical performance is an important indicator that reflects current and predicts future health. In this study, we examined the association of alcohol use and depression with grip strength a national sample of middle aged and older Chinese adults. Methods: We used the baseline data from the China Health and Retirement Longitudinal Study (CHARLS) and constructed a multivariate linear regression using SAS 9.4 to examine the independent association of alcohol use (never, former, moderate, and at-risk drinkers) and depression with grip strength controlling for socio-economic factors and domestic partner status. Results: The study population consisted of 12,488 Chinese adults（mean age 59）. The prevalence of ever drinking during lifetime and current at-risk drinking (>14 standard drinks [one standard drink contains 14 grams of pure alcohol] per week) in this population was 25.7% and 15.2% respectively. 28.4% of the study population had depression. Compared with never drinkers, moderate and at-risk alcohol use were independently associated with better grip strength (P<0.0001). Depression was independently negatively associated with grip strength (P<0.0001). Conclusions: We found that current alcohol use might be protective of grip strength while depression might be detrimental to grip strength among middle-aged adults. However, the underlying mechanism is unclear. Given the negative impact of alcohol and depression on adults’ overall health, clinicians should assess alcohol use and depression in middle-aged and older patients using validated tools and provide resources. Clinicians should counsel patients that if depression is not managed, patients may suffer from depression associated health consequences such as declined grip strength.

